# HPV vaccination is fundamental for reducing or erradicate penile cancer | *Opinion: NO*

**DOI:** 10.1590/S1677-5538.IBJU.2018.05.03

**Published:** 2018

**Authors:** Paulo Ornellas, Antonio Augusto Ornellas

**Affiliations:** 1 Departamento de Patologia, Universidade do Estado do Rio de Janeiro, Rio de Janeiro, RJ, Brasil; 2 Departamento de Urologia, Instituto Nacional do Câncer (INCA), Rio de Janeiro, RJ, Brasil; 3 Instituto de Pós-Graduação Médica Carlos Chagas, Rio de Janeiro, RJ, Brasil

**Keywords:** Human papillomavirus, Penile Neoplasms, Vaccination, Male

Human papillomavirus (HPV) is a DNA virus that presents tropism for epithelial cells, causing infections of the skin and mucous membranes. It is transmitted by direct contact of a healthy skin or mucosa with an affected skin or mucosa. Until now, more than 200 types of HPVs have been discovered (1). Approximately 30 types infect the anal and genital mucosa. Types that can also be detected in the oral mucosa are classified according to risk of causing lesions or their potential for malignancy. Such as “low risk” are included types 6 and 11 (more incidents) and as “high risk” the types 16, 18, 31, 33, 35, 39, 45, 51, 52, 56, 58, 59 and 66 (2). Human papillomavirus (HPV), a sexually transmitted infection, is responsible for 99.7% of cases of cervical cancer (3) and 530,000 new cases of cervical cancer globally every year (4). In addition, HPV is also responsible for some head and neck cancers, penile cancers and the majority of anal cancers (5). The incidence of these cancers is on the rise. Globally, HPV types 16 and 18 are responsible for 38,000 (85%) new cases of head and neck cancers and 35,000 (87%) cases of anal cancers (4).

The main focus of global vaccine programs has been prevention of cervical cancer, through prevention of oncogenic HPV infection, the necessary cause of squamous and glandular cervical carcinomas (6, 7). The most robust available data regarding HPV vaccines regards cervical intraepithelial neoplasia and cervical cancer. A recent 10-year review and meta-analysis of several randomized controlled trials (RCT) cited efficacy from 89.8-100% in a follow-up of 34.9 months to 9.4 years (8). The most common adverse event was pain at the injection site and any serious adverse events were not determined to be vaccine-related (8). Another 10-year review found similar efficacy, specifically of the bivalent and 9-valent vaccines, again, in CIN2+ lesions (9). However, regarding male HPV cases, a significant concern is the low rate of seroconversion after natural infection (10). In addition, it has been suggested that HPV antibody seropositivity does not provide significant immunity to future infections like it does in women (11). Fortunately, the quadrivalent HPV vaccine has been shown to be highly immunogenic in men age 16 to 26, with seroconversion by month 7, remaining elevated even at 36 months, with titers comparable to those in women (12). In Sweden, the current HPV Immunization Program includes only young females, and coverages above 50% in such programs are suggested to give herd immunity (13, 14). Since the introduction of the HPV vaccine in Sweden, the incidence of anogenital warts has decreased among both the vaccinated female population and the unvaccinated male population, suggesting that herd immunity has been achieved (15, 16). However, among males having sex with males, herd immunity has not yet been proven, which is why a recent modeling study recommended targeted prevention strategies to reach this population (17).

Penile cancer is a heterogeneous disease with respect to HPV infection, with the association with penile infection dependent on the histology (18, 19). It is uncertain whether cancers involving HPV infection have better survival profiles than cancers without HPV infection. In a study with 82 penile cancer patients, 30.5% of tumors had HPV DNA, with HPV 16 being the most prevalent. This study demonstrated no association between HPV negative and positive patients when considering lymph node metastasis (P=0.386) and 10-year survival rate (68.4% vs. 69.1%; P=0.83) (20). In another study with 29 patients with invasive squamous cell carcinoma of penis (SCCP), 31% of tumors had either HPV-16 or HPV-18 DNA. This study found no difference between HPV negative and positive patients in terms of nodal metastasis or survival even after adjustment control for tumor stage (21). However, these results differ from another which examined HPV status as a prognostic indicator in 171 penile cancer patients. In this study, high-risk HPV DNA was found in 29% of tumors, with 76% containing HPV-16. High-risk HPV was associated with improved 5-year disease-specific survival (78% vs. 93%; P=0.03). Additionally, high-risk HPV was an independent predictor of disease specific mortality in multivariate analysis [hazard ratio (HR), 0.14; 95% CI, 0.03-0.63; P=0.01] (22). Regarding all these studies, we can notice that the presence HPV DNA in SCCP is not much higher than 30 %. It means that approximately 70% of patients with SCCP will still have the disease even if all males received the vaccine. SCCP have a low association with HPV, whereas warty/basaloid cancers are strongly associated with HPV (23). In addition, preliminary data indicate a prevalence of 54.6% of HPV cases among the Brazilian population aged 16 to 25 years, 38.4% of which are of high risk for the development of cancer (24). he infection can manifest itself in two ways: clinical and subclinical and it is estimated that only about 5% of people infected with HPV will develop some form of manifestation (25). As penile cancer accounts for only 2.7% of male malignancies in Brazil (26), we can roughly estimate that few patients with HPV will develop penile cancer. Among HPV positive patients, those with high-risk HPV would be more likely to develop penile cancer.

Therefore, the male public HPV vaccination is a good measure to prevent not just cervical cancer but some head and neck cancers, some penile warts and the majority of anal cancers. However, it is not clear if it will be enough to eradicate or reduce the prevalence of penile cancer. Increased patient education along with prevention strategies such us condom use, hygienic measures, smoking cessation, and avoidance of chronic inflammatory states can have considerable impact on pathogenesis of pre-cancerous lesions of the penis. Although, the adoption of HPV vaccination has led to some success in female HPV-related cancers, the results are yet to be elucidated in the male population. It is necessary further long-term studies to declare that HPV vaccination is effective against SCCP.
